# Heteroplasmic and homoplasmic m.616T>C in mitochondria tRNA^Phe^ promote isolated chronic kidney disease and hyperuricemia

**DOI:** 10.1172/jci.insight.157418

**Published:** 2022-06-08

**Authors:** Chengxian Xu, Lingxiao Tong, Jia Rao, Qing Ye, Yuxia Chen, Yingying Zhang, Jie Xu, Xiaoting Mao, Feilong Meng, Huijun Shen, Zhihong Lu, Xiaohui Cang, Haidong Fu, Shugang Wang, Weiyue Gu, En-Yin Lai, Min-Xin Guan, Pingping Jiang, Jianhua Mao

**Affiliations:** 1 Department of Nephrology, The Children’s Hospital, Zhejiang University School of Medicine and National Clinical Research Center for Child Health, Hangzhou, China.; 2 Department of Nephrology, Children’s Hospital of Fudan University, National Pediatric Medical Center of China, Shanghai, China.; 3 Zhejiang Key Laboratory for Neonatal Diseases, The Children’s Hospital of Zhejiang University School of Medicine, Hangzhou, China.; 4 Department of Rehabilitation Medicine, Children’s Hospital of Chongqing Medical University, National Clinical Research Center for Child Health and Disorders, Ministry of Education Key Laboratory of Child Development and Disorders, Chongqing Key Laboratory of Pediatrics, Chongqing, China.; 5 Institute of Genetics and Department of Human Genetics, Zhejiang University School of Medicine, Hangzhou, China.; 6 Chigene (Beijing) Translational Medical Research Center Co. Ltd., Yizhuang, Beijing, China.; 7 The Department of Physiology, Zhejiang University School of Medicine, Hangzhou, China.

**Keywords:** Nephrology, Chronic kidney disease, Mitochondria

## Abstract

Inherited kidney diseases are the fifth most common cause of end-stage renal disease (ESRD). Mitochondrial dysfunction plays a vital role in the progression of inherited kidney diseases, while mitochondrial-transfer RNA (mt-tRNA) variants and their pathogenic contributions to kidney disease remain largely unclear. In this study, we identified the pathogenic mt-tRNA^Phe^ 616T>C mutation in 3 families and documented that m.616T>C showed a high pathogenic threshold, with both heteroplasmy and homoplasmy leading to isolated chronic kidney disease and hyperuricemia without hematuria, proteinuria, or renal cyst formation. Moreover, 1 proband with homoplamic m.616T>C presented ESRD as a child. No symptoms of nervous system evolvement were observed in these families. Lymphoblast cells bearing m.616T>C exhibited swollen mitochondria, underwent active mitophagy, and showed respiratory deficiency, leading to reduced mitochondrial ATP production, diminished membrane potential, and overproduction of mitochondrial ROS. Pathogenic m.616T>C abolished a highly conserved base pair (A31-U39) in the anticodon stem-loop which altered the structure of mt-tRNA^Phe^, as confirmed by a decreased melting temperature and slower electrophoretic mobility of the mutant tRNA. Furthermore, the unstable structure of mt-tRNA^Phe^ contributed to a shortage of steady-state mt-tRNA^Phe^ and enhanced aminoacylation efficiency, which resulted in impaired mitochondrial RNA translation and a significant decrease in mtDNA–encoded polypeptides. Collectively, these findings provide potentially new insights into the pathogenesis underlying inherited kidney disease caused by mitochondrial variants.

## Graphical abstract

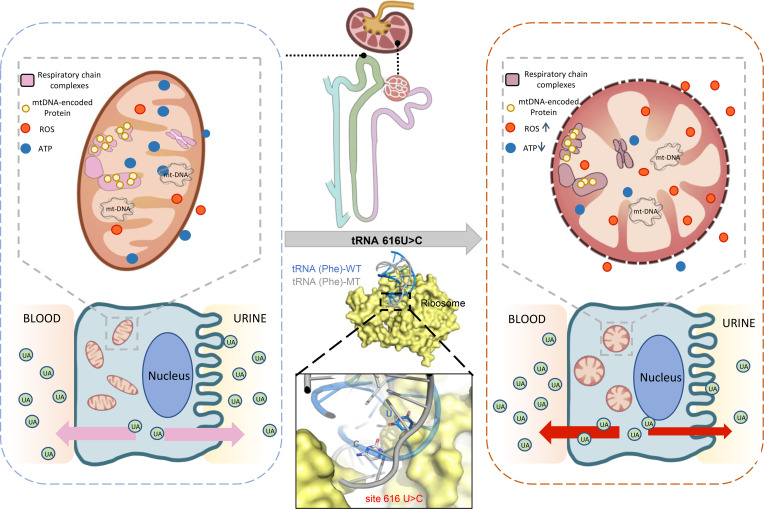

## Introduction

Inherited kidney diseases are the fifth most common cause of end-stage renal disease (ESRD) after diabetes, hypertension, glomerulonephritis, and pyelonephritis. At least 10% of adults and nearly all children who progress to renal replacement therapy have inherited kidney disease (1, 2). As the kidney is the second most important organ after the heart in oxygen consumption and mitochondrial abundance (3–5), mitochondrial dysfunction or defects in mitochondrial dynamics are thought to be involved in a variety of kidney diseases (6–8). However, the impact of mitochondrial dysfunction on the development of kidney disease remains limited.

Kidney diseases caused by mitochondrial dysfunction, involving mitochondrial resident protein encoded by both nuclear and mitochondrial genes, are usually characterized by defects in tubular or glomerular tissues, including tubulopathies, tubulointerstitial nephritis, steroid resistant nephrotic syndrome, and/or cystic renal disease (7). One important mitochondrial genetic cause is the m.3243A>G mutation of mt-tRNA^leu^, manifesting in proteinuria and focal segmental glomerular sclerosis (FSGS) (9). FSGS has also been reported to be caused by mt-tRNA^Asn^ (m.5728A>G) with neurological involvement (10) or mt-tRNA^Tyr^ (m.5843 A>G) with dilated cardiomyopathy (11). Additionally, a defect in the distal convoluted tubule with hypomagnesaemia was due to a mt-tRNA^Ile^ (m.4291T>C) variant and presented with increased excretion of urinary Mg^2+^ and decreased excretion of urinary Ca^2+^ (12). Recently, patients with tubulointerstitial kidney disease and/or epilepsy were found to harbor variant mt-tRNA^Phe^, including homoplasmic m.616T>C (13, 14). However, the molecular mechanisms by which these variants exert effects remain to be elucidated. Here, we identified both homoplasmic and heteroplasmic m.616T>C associated with isolated chronic kidney disease (CKD) and hyperuricemia in 3 Chinese families. Along with a detailed clinical evaluation, we investigated the role of the mt-tRNA^Phe^ variant using cell models to better understand the pathogenesis of this CKD and related hyperuricemia.

## Results

### Clinical manifestations and identification of both homoplasmic and heteroplasmic m.616T>C.

**Figure 1:**
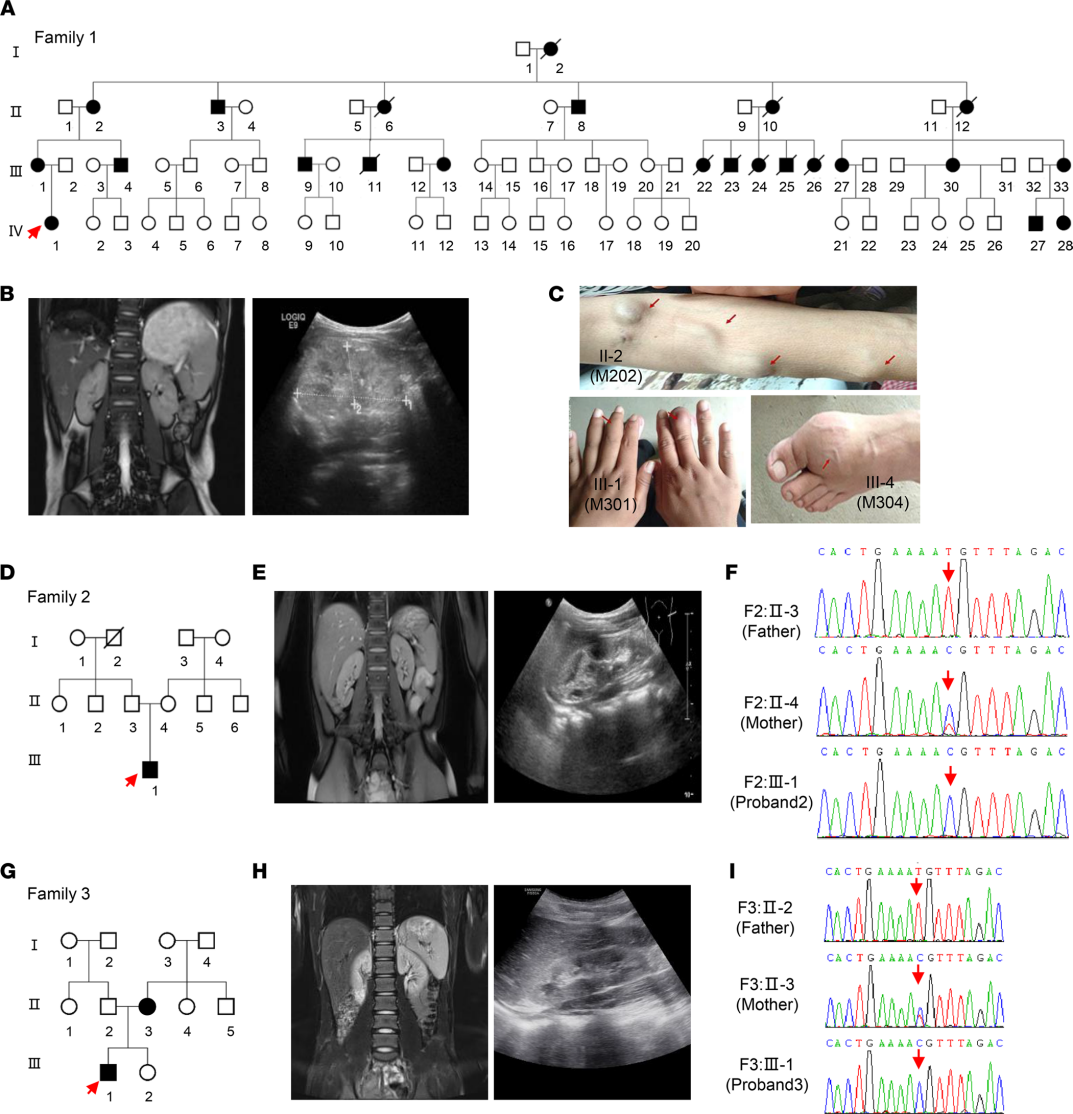
Pedigrees showing the inheritance of the mt-tRNA^Phe^ 616T>C variant. (**A**) Pedigree of family 1 carrying the homoplasmic m.616T>C variant (MT-TF). (**B**) MRI scans and B ultrasound of the proband in family 1 showing decreased kidney size in proband 1. Bar: 10 cm. (**C**) Tophi and swollen joint phenotypes in several affected patients in family 1 (II-2, III-1, and III-4). (**D**) Pedigree of family 2 carrying the m.616T>C variant. (**E**) MRI scans and B ultrasound of the proband in family 2 showing abnormal changes. (**F**) Homoplasmic and heteroplasmic m.616T>C identified in family 2 by Sanger-Seq. (**G**) Pedigree of family 3 carrying the m.616T>C variant. (**H**) Abdominal CT scan and B ultrasound of proband 3 showing abnormal changes. (**I**) Homoplasmic and heteroplasmic m.616T>C identified in family 3 by Sanger-Seq. Squares indicate males; circles indicate females; solid indicate patients; and arrows indicate probands.

Family 1 (F1) originated from Anhui Province in southern China (Figure 1A). Proband 1 (M401, F1: IV-1), a girl aged 6 years and seven months, was admitted to the hospital because of frequently recurrent swelling and pain in the hands and feet. Routine blood tests revealed renal insufficiency (serum creatinine [Scr], 257 μmol/L; CCR 10.8 mL/min), hyperuricemia (serum uric acid 589.5 μmol/L, urine uric acid excretion 6.9%), and anemia (Hb 85 g/L). No hematuria or proteinuria were found in urine testing, and 24-hour urine protein excretion was 73.6 mg. Her estimated glomerular filtration rate (eGFR) was 13 mL/min (Table 1). An abdominal ultrasound B-scan revealed diffuse changes in both kidneys (Figure 1B). MRI demonstrated the following: (a) both kidneys had decreased in size (the left kidney was 6.6*2.8 cm and the right was 6.3*2.7 cm) and showed no cysts (Figure 1B); and (b) there was no obvious abnormal finding in the cerebral MRI scan. Renal biopsy was refused by her mother because of the girl’s severe nephropathy. After administering febuxostat, calcium carbonate, and calcitriol, renal function continuously deteriorated; she finally received renal transplantation at another hospital 1 year after administration. Her glomerular and tubular functions have remained normal since then.

Laboratory tests revealed that the mother (F1: III-1) also suffered renal insufficiency (Scr, 286 μmol/L) with an eGFR value of 18 mL/min, and the father (F1: III-2) had a normal index (Table 1). After interviewing other family members, 23 of 31 matrilineal relatives reported CKD and/or hyperuricemia with a wide range in severity throughout 4 generations (Figure 1C). Further, none of the affected individuals among the relatives presented nervous system dysfunction such as epilepsy or seizures. Affected fathers did not transmit traits to their offspring, indicating that it was inherited via the mitochondrial genome. Therefore, we set out to analyze the mitochondrial genome of matrilineal relatives and patrilineal members. The entire mtDNA of the proband was analyzed directly by Sanger-Seq (Supplemental Figure 1 and Supplemental Table 1; supplemental material available online with this article; https://doi.org/10.1172/jci.insight.157418DS1). A total 39 variants belonging to Eastern Asian haplogroup D4 were identified (15), in which a pathogenic variant, m.616T>C, in mt-tRNA^Phe^ was found with a frequency of 0.002% (GenBank), as well as 38 known polymorphisms (MitoMap, www.mitomap.org/foswiki/bin/view/MITOMAP/). To determine whether the m.616T>C variant was evident through homoplasmy or heteroplasmy, fragments spanning mt-tRNA^Phe^ were amplified by PCR and subsequently digested with Acl I since m.616T>C showed a site for this restriction enzyme. There was no detectable WT DNA in the matrilineal relatives (Supplemental Figure 1), indicating homoplasmic m.616T>C in the matrilineal relatives, similar to other families (14). In addition to peripheral blood, urine and skin tissue samples from proband 1 were collected, and high-throughput sequencing confirmed the homoplasmic variant in these tissues (Supplemental Figure 1). Finally, 11 living maternal individuals who carry the homoplasmic m.616T>C variant were identified, but this variant was absent in patrilineal members who served as the controls in the family. Additionally, mitochondrial genome polymorphisms of the proband’s father belonged to Eastern Asian haplogroup G2 and showed no novel or pathogenic variants (15).

Family 2 (F2) originated from Jiangxi Province in China (Figure 1D). A 7-year-old boy, proband 2 (F2: III-1) suffered from transient salivation, aphasia, and left upper extremity weakness at the age of 2 years, and recovered sooner. He suffered from abdominal pain and vomiting at this onset, manifesting with Scr of 125.5 μmol/L and uric acid concentration of 785.0 μmol/L. His 24-hour urine protein was 40 mg, and no hematuria was found. Abdominal ultrasound and MRI scans showed abnormal changes in both kidneys without cysts (the size of the left kidney was 7.2*2.9 cm and that of the right kidney was 7.2*3.0 cm) (Figure 1E). No obvious abnormal finding was observed in cerebral MRI, magnetic resonance venography (MRV), and/or magnetic resonance angiography (MRA). His eGFR value was 40 mL/min while the total GFR was 23.5 mL/min detected by diethylenetriamine pentacetate (DTPA) tests with a value of 11.9 mL/min for the left and of 11.6 mL/min for the right kidney (Supplemental Figure 2). Sanger-Seq and high-throughput sequencing led to the identification of the homoplasmic m.616T>C variant in proband 2 (F2: III-1) with a haplogroup F2 (Figure 1F and Supplemental Table 1). However, a heteroplasmic m.616T>C was identified by Sanger-Seq in the peripheral blood lymphocyte (PBL) sample of the mother (F2: II-4), and confirmed to a loan of 68.21% by high-throughput sequencing (Supplemental Figure 2), whose routine blood tests for renal function was normal but who self-reported that she had nephrolithiasis. The mother refused the collection of urine samples for use in testing the variant heteroplasmy.

Family 3 (F3) also originated from Jiangxi province in China (Figure 1G). Proband 3 (F3:III-1) was diagnosed with CKD (G3) and hyperuricemia at age 12 during a routine physical examination. The Scr of proband 3 was 108.0 μmol/L and the serum uric acid was 783.0 μmol/L. No proteinuria or hematuria was found in urine testing. Both abdominal ultrasound and MRI scan revealed small-sized kidneys (the left kidney was 8.4*4.0 cm and the right kidney was 7.5*3.4 cm) (Figure 1H). DTPA tests indicated impaired function of kidneys (17.9 mL/min for the left kidney and 15.9 mL/min for the right kidney). Using whole mitochondrial genome sequencing, homoplasmic m.616T>C was identified in proband 3 (Supplemental Figure 2), which was confirmed by Sanger-Seq with haplogroup B5 (Figure 1I and Supplemental Table 1). However, heteroplasmic m.616T>C was identified from a sample of the mother (F3: II-3), and the variant presented various degrees of heteroplasmy in samples from different tissues: 76.27% in PBL, 89.85% in saliva, and 94.5% in urine. Blood tests revealed that the mother suffered from CKD (G2) with Scr of 92.1 μmol/L, blood urea nitrogen (BUN) of 10.76 mmol/L, and an eGFR of 60.2 mL/min. Additionally, heteroplamic m.616T>C expression varied in different tissues in an asymptomatic younger sister (8 years old) with samples that showed the variant at 55.17% in PBL, 77.25% in saliva, and 87.26% in urine (Supplemental Figure 2).

### Kidney injury and abnormal mitochondrial morphology in cells.

**Figure 2:**
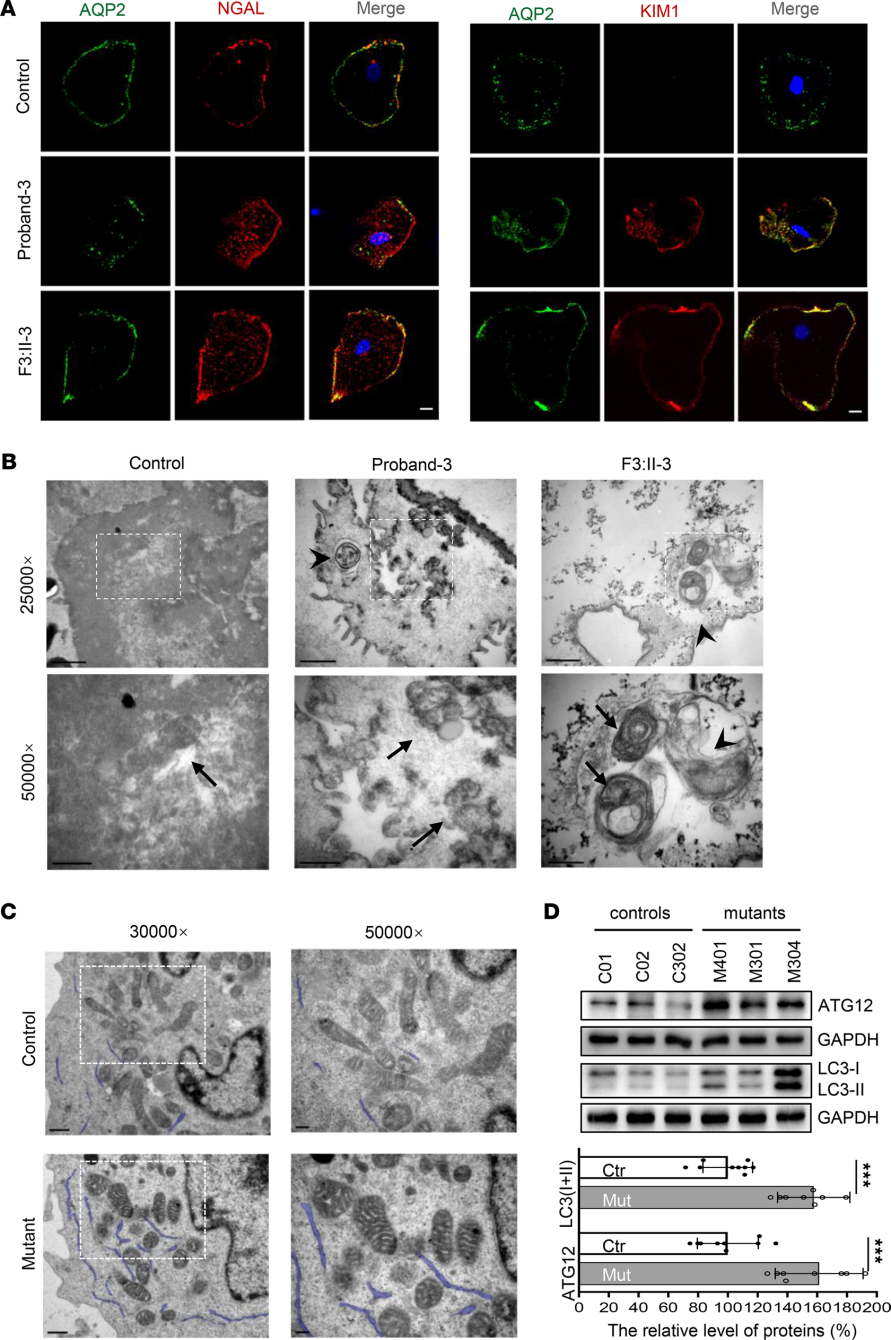
Kidney injury and abnormal mitochondrial morphology in cells. (**A**) Immunofluorescence analysis of KIM1 and NGAL expression in tubular epithelial cells. AQP2, a water channel protein located in the kidney collecting tubule. Bar: 10 μm (**B**) Mitochondrial morphology in tubular epithelial cells by TEM. Arrows, mitochondria; arrowhead, autophagosome. Bar: 1 μm (upper), 0.5 μm (lower). (**C**) Mitochondrial morphology in lymphoblasts by TEM. Bar: 0.5 μm (left), 0.2 μm (right). (**D**) Mitophagy analysis by Western blotting with Abs against ATG12 and LC3I/II and quantification analysis. The data are expressed as the means ± SD with 3 independent experiments. Student’s *t* test was performed to determine statistically significant differences. ***P < 0.0001.

As the biopsy or nephrectomy samples were unavailable, we collected urine samples from proband 3 and the mother (F3: II-3) to examine whether the tubular epithelial cells that harbored homoplasmic or heteroplasmic m.616T>C would present features associated with kidney injury. As expected, higher expressions of kidney injury molecule 1 (KIM1) and neutrophil gelatinase-associated lipocalin (NGAL) were evidenced by immunofluorescence analysis in patients’ samples compared with the controls (Figure 2A). As abnormal morphology of mitochondria was described during decline in renal function previously (16), transmission electron microscopy (TEM) analysis was performed in both tubular epithelial cells and lymphoblastoid cells derived from patients and controls. As shown in Figure 2, B and C, swollen mitochondria and patchy cristae were present in patients’ cells, and some autophagosomes were visualized in epithelial cells from proband 3 and the mother. Moreover, increased expression of ATG12, LC3-I, and LC3-II was found in mutant cells (Figure 2D), confirming that the swollen mitochondria was due to m.616T>C variant–activated mitophagy, which was activated to prevent cell death. However, no significant alteration was detected in mitochondrial-shaping proteins, neither OPA1 nor MFN1 (Supplemental Figure 3).

### The m.616T>C variant caused mitochondrial dysfunction without mtDNA depletion.

**Figure 3:**
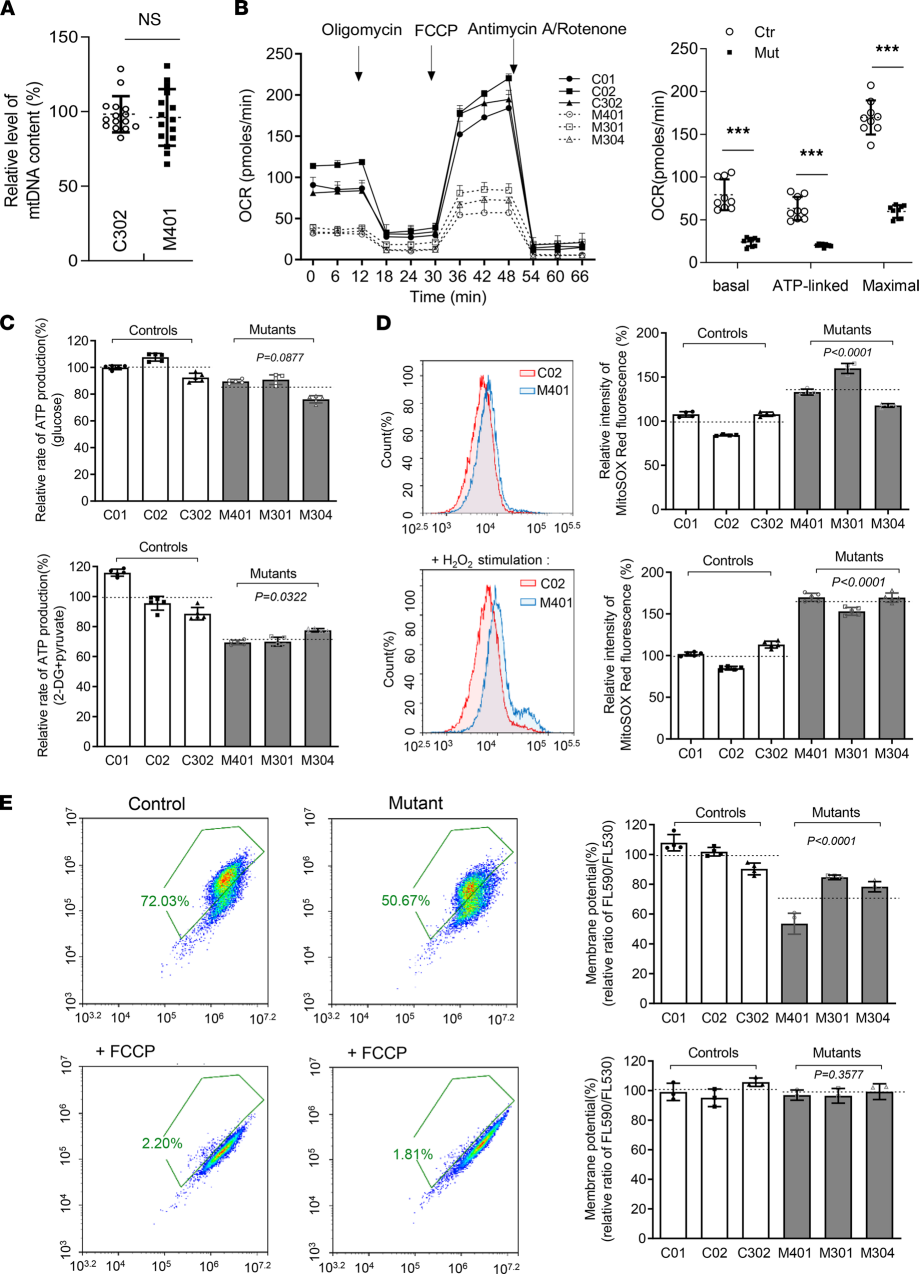
Mitochondrial dysfunction in mutant cells. (**A**) Relative level of mtDNA content. qPCR was performed to analyze the expression of the mitochondrial 16S rRNA gene and nuclear 18S rRNA gene (*n* = 15). (**B**) The OCR with after cells were treated at the indicated times with different inhibitors: oligomycin (1 μM), FCCP (0.8 μM), rotenone (1 μM), and antimycin A (5 μM). Quantification of the basal OCR, ATP-linked OCR, and maximal OCR in mutant and control cell lines. The average values of 3 measurements for each cell line are shown. (**C**) Cellular and mitochondrial ATP production was detected by luciferase assay (*n* = 5). Cells were incubated with 10 mM glucose or 5 mM 2-deoxy-D-glucose plus 5 mM pyruvate to determine ATP generation under mitochondrial ATP synthesis. (**D**) Mitochondrial ROS were measured by flow cytometric analysis, using a MitoSOX Red Superoxide Indicator with or without H_2_O_2_ stimulation (*n* = 5). (**E**) MMP measurements using a fluorescence probe JC-10 assay system with (*n* = 3) or without (*n* = 4) 10 μM of FCCP stimulation. The relative ratio of JC-10 fluorescence intensities at Ex/Em = 490/530 nm and 490/590 nm. The calculations were based on 3 independent experiments. The data are expressed as the means ± SD. Student’s *t* test was performed to determine statistically significant differences. ***P < 0.0001.

Point variants of mt-tRNAs are the primary cause of mitochondrial dysfunction. To exclude the impact of mtDNA depletion on mitochondrial function, we determined the mtDNA copy number with real-time PCR and found no change in blood samples from proband 1 in relation to that of her father (Figure 3A). Mitochondrial oxidative phosphorylation (OXPHOS) activity was measured by the oxygen consumption rate (OCR) obtained with an XF-96 Extracellular Flux Analyser. The mutant cells exhibited lower OCRs with a notable decrease in basal (30.45%, *P* < 0.0001), ATP-linked (31.91%, *P* < 0.0001), and maximal OCRs (35.17%, *P* < 0.0001) relative to the mean value in the control cells (Figure 3B). Consistently, diminished ATP production in mitochondria was found in mutant cells at 71.9% (*P* < 0.001) that of controls (Figure 3C). The production of mitochondrial ROS in mutant cells reached 136.89% (*P* < 0.0001) and increased to 164.20% (*P* < 0.0001) with H_2_O_2_ stimulated, as tracked by a MitoSOX indicator (Figure 3D). Finally, the mitochondrial membrane potential (MMP) in mutant cells, as an indispensable component in the process of energy storage during OXPHOS, was 72.16% (*P* < 0.0001) that of the mean values measured in the control cell lines (Figure 3E). Together, these data indicate that the point variant m.616T>C resulted in mitochondrial dysfunction without mtDNA depletion.

### The m.616T>C variant altered the conformation and stability of mt-tRNA^Phe^.

**Figure 4:**
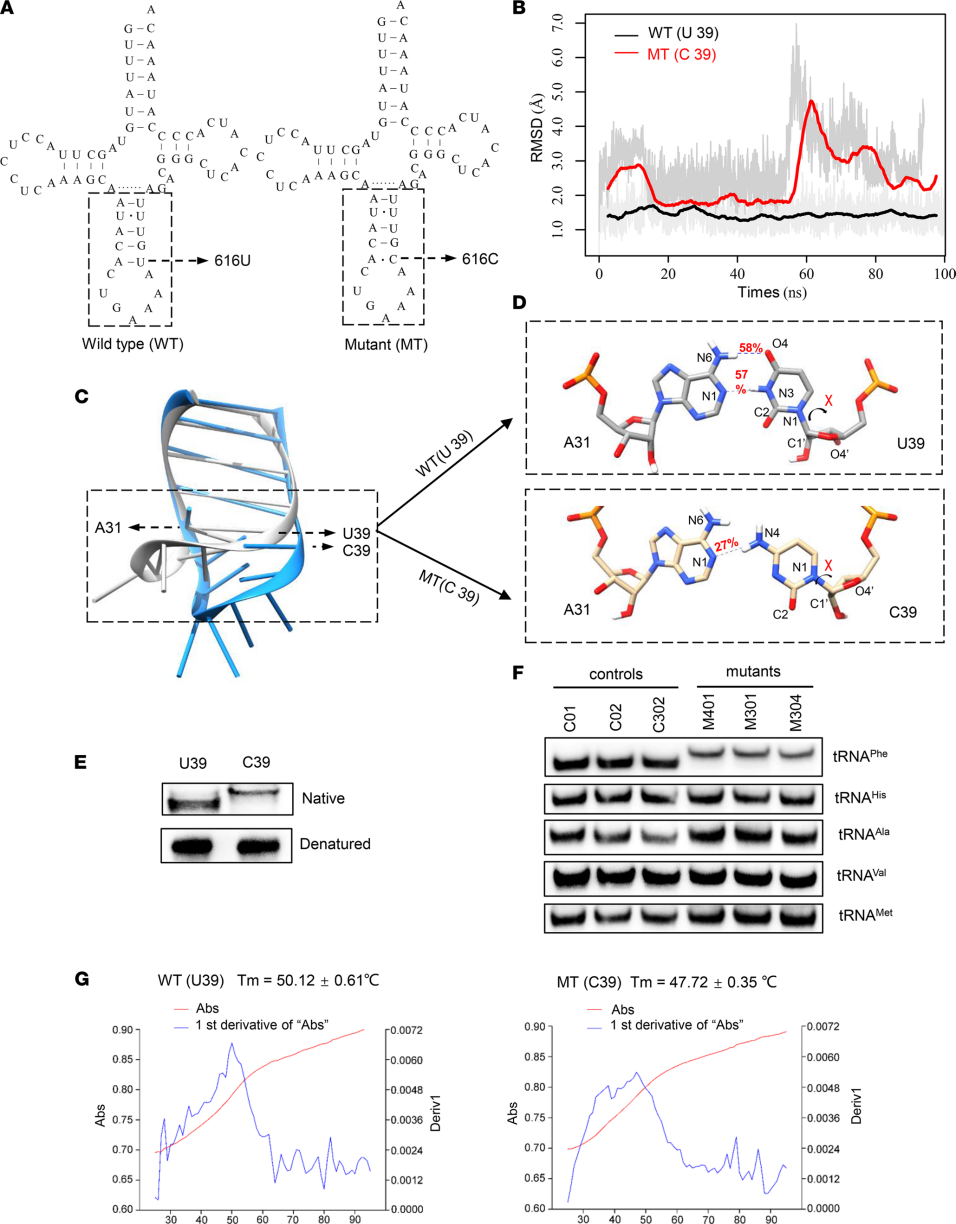
MD simulation analysis and mt-tRNA^Phe^ stability assay. (**A**) Cloverleaf structure of human mt-tRNA^Phe^. The arrow indicated the location of the m.616T>C variant. Nts in the dashed box in the ASL of mt-tRNA^Phe^ were used for MD simulation analysis. (**B**) Time evolution of the RMSD values of all backbone atoms in the ASL in WT (black curve) and mutant (MT) (red curve) tRNA^Phe^. (**C**) Conformational differences in the ASL in WT (grey) and mutant (dodger blue) mt-tRNA^Phe^. (**D**) Hydrogen bonds (blue dashes) between A31 and U/C39 in WT (grey) and mutant mt-tRNA^Phe^ (wheat). The hydrogen bonds are shown in red. (**E** and **F**) A tRNA mobility shift assay using the in vitro transcripts of WT (U39) and mutant (C39) mt-tRNA^Phe^ in native gel with a denaturing gel control experiment performed in parallel **E**, and using RNA from control and mutant cell lines, which were hybridized with the DIG-labeled oligonucleotide probes specific for mt-tRNA^Phe^, mt-tRNA^His^, mt-tRNA^Ala^, mt-tRNA^Val^, and mt-tRNA^Met^, respectively, for native gel assays. (**G**) Thermal stability of WT (U39) and mutant (C39) mt-tRNA^Phe^. Absorbance of WT and MT measured at 260 nm with heating from 25°C–95°C at a rate of 1°C per minute (red curves). The first derivative (dA/dt) of the temperature curves is shown to highlight the Tm value transitions (blue curves). The calculations were based on 3 independent experiments.

According to the classic cloverleaf structure of human mt-tRNA^Phe^, the uridine (U) to cytosine (C) transition at position 39 (U39) alters the original base pair A31-U39 in the anticodon stem-loop (ASL) region, as shown in Figure 4A (17). To investigate whether m.616T>C disrupts the structure and stability of mt-tRNA^Phe^, we performed molecular dynamic (MD) simulations to assess the impact of the mutation on the tertiary structure of mt-tRNA^Phe^. The ASL (17 nt) structure and dynamics of WT and mutant mt-tRNA^Phe^ were evaluated by 100-ns all-atom MD simulations. As shown in Figure 4B, the root mean square deviation (RMSD) curve of the mutated ASL fluctuated to a greater degree than that of its WT counterpart, suggesting that the mutated stem-loop is more unstable than its WT counterpart. Superposing the structures at the end of the simulations revealed an apparent conformational difference between the WT and mutant ASL (Figure 4C). In the WT mt-tRNA^Phe^, U39 forms a canonical base pair with A31 through 2 hydrogen bonds that show 58% and 57% occupancy (Figure 4D). In contrast, the newly formed interaction between A31 and C39 in the mutant mt-tRNA^Phe^ was decreased to 1 hydrogen bond with a lower occupancy of 27%. These results strongly indicated that the disruption of the A31-U39 base pair in the anticodon stem of mt-tRNA^Phe^ reduced the stability of mt-tRNA^Phe^. Therefore, we performed a tRNA mobility shift assay to confirm the subtle conformational changes (Figure 4E) as previously described (18). The in vitro mutant (C39) transcript migrated slower than the WT (U39) transcript in a native PAGE gel, indicating that C39 loosened the mt-tRNA^Phe^ structure. In contrast, no different migratory pattern between the U39 and C39 transcripts under denaturing conditions was observed. In addition, the in vivo mt-tRNA^Phe^ from mutant cell lines bearing m.616T>C moved much more slowly in the native gel than that of the controls, and no different migratory patterns were evident between mt-tRNA^His^, mt-tRNA^Ala^, mt-tRNA^val^, and mt-tRNA^Met^ in either the mutant or control cell lines (Figure 4F), corroborating evidence that m.616T>C affects the structure and stability of mt-tRNA^Phe^. Additionally, we measured the melting temperature (Tm) to verify that the C39 transcript showed less stability than the WT (U39) transcript. Consistent with the prediction made through MD analysis, the Tm value of the C39 transcript at 47.72°C was lower than that of U39, which was 50.12°C for the WT transcript (Figure 4G). Taken together, these data demonstrated that the m.616T>C variant changed the conformation of mt-tRNA^Phe^ and weakened its attenuated stability.

### The m.616T>C variant decreased the level of steady-state mt-tRNA^Phe^ and enhanced aminoacylation.

**Figure 5:**
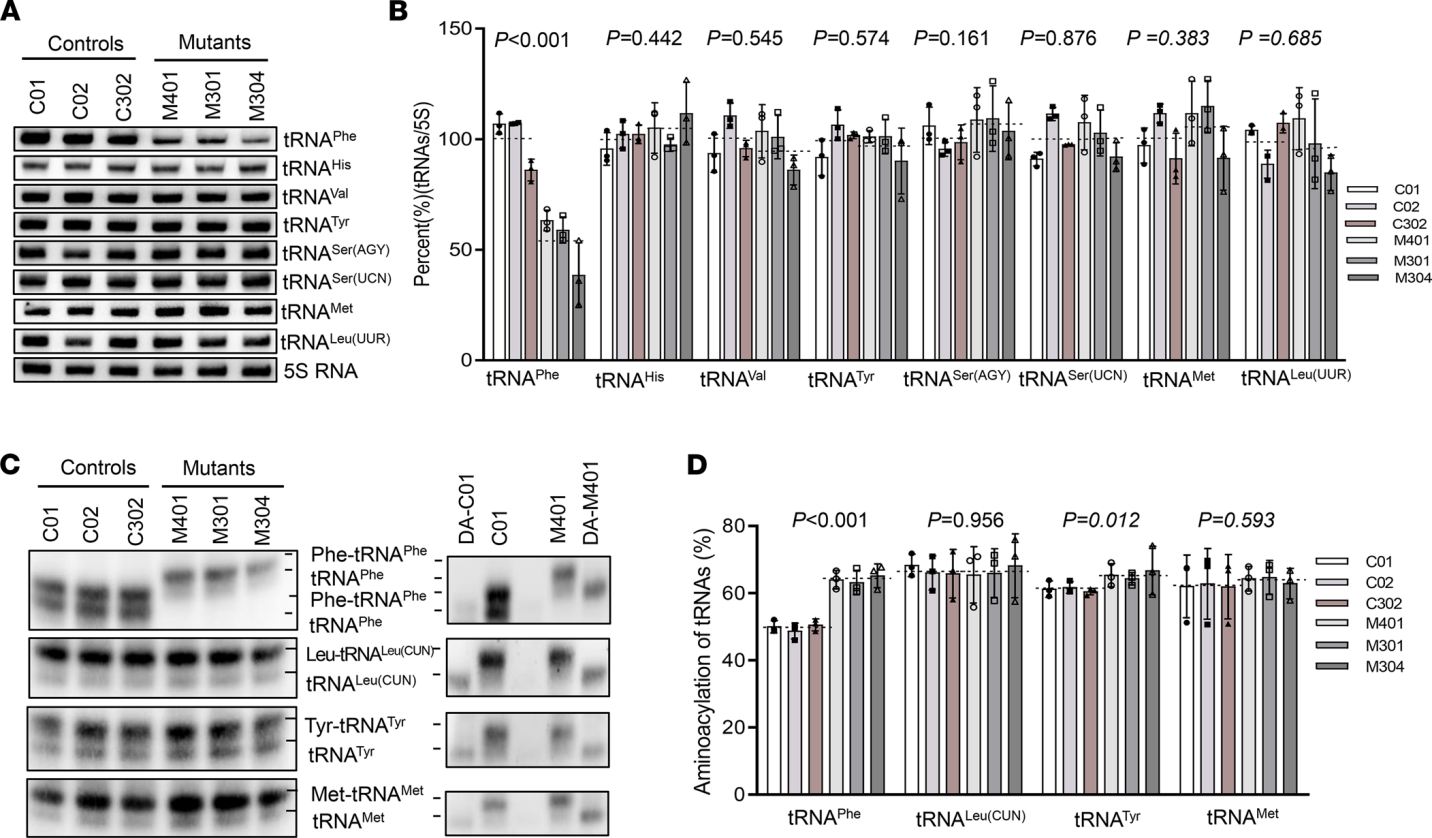
Northern blot analysis of mt-tRNA under denaturing conditions. (**A**) Northern blot analysis of mt-tRNA under denaturing conditions. DIG-labeled oligodeoxynucleotide probes specific for the mt-tRNA^Phe^, mt-tRNA^His^, mt-tRNA^Val^, mt-tRNA^Ser(AGY)^, mt-tRNA^Met^, mt-tRNA^Leu(UUR)^, mt-tRNA^Ser^ ^(UCN)^, mt-tRNA^Tyr^, and 5S rRNA as a loading control. (**B**) Quantification of mt-tRNA^Phe^ levels. (**C**) Aminoacylation assays. RNA was hybridized with specific probes of mt-tRNA^Phe^, mt-tRNA^Leu(CUN)^, and mt-tRNA^Tyr^, and deacylation treatment was performed in parallel to distinguish charged and uncharged tRNAs. DA, deacylated. (**D**) The proportion of aminoacylated mt-tRNA^Phe^ in control and mutant cell lines. The calculations were based on 3 independent experiments. The data are expressed as the means ± SD (*n* = 3). Student’s *t* test was performed to determine statistically significant differences.

To understand the influence of the m.616T>C on tRNA metabolism, we investigated the transcription of mt-tRNA^Phe^ ex vivo. In addition to a mt-tRNA^Phe^ probe, DIG-labeled oligodeoxynucleotide probes for detecting mt-tRNA^His^, mt-tRNA^Val^, mt-tRNA^Ser(AGY)^, mt-tRNA^Met,^ and mt-tRNA^Leu(UUR)^, representatives of the heavy-strand (H-strand) transcription unit, and mt-tRNA^Ser(UCN)^ and mt-tRNA^Tyr^ derived from light-strand (L-strand) transcript were hybridized for Northern blotting analysis. As illustrated in Figure 5, A and B, the average steady-state levels of mt-tRNA^Phe^ in the mutant cells decreased significantly, by approximately 53.6% (*P* < 0.0001) that of the average values of 3 control cell lines after normalization to the corresponding 5S rRNA. However, there was no significant difference in the average levels of other H-strand transcript or L-strand transcript between the mutant and control groups. Aminoacylation was then evaluated using an acidic urea PAGE system to separate charged and uncharged tRNAs (Figure 5, C and D), and the nonaminoacylated tRNAs were confirmed by diacylation treatment in parallel. Strikingly, the efficiency of aminoacylated tRNA^Phe^ harboring C39 was enhanced, increasing from a mean value of 49.8% in the controls to 64.1% (*P* < 0.0001) in the mutants. However, the levels of aminoacylation of mt-tRNA^Met^, mt-tRNA^Leu(CUN)^, and mt-tRNA^Tyr^ in mutant cells were comparable with those in the control cells. It seemed that both the decreased level of steady-state mt-tRNA^Phe^ and its increased aminoacylation contributed to the translation of mitochondrial polypeptides.

### The m.616T>C variant decreased the levels of mitochondrial proteins.

**Figure 6:**
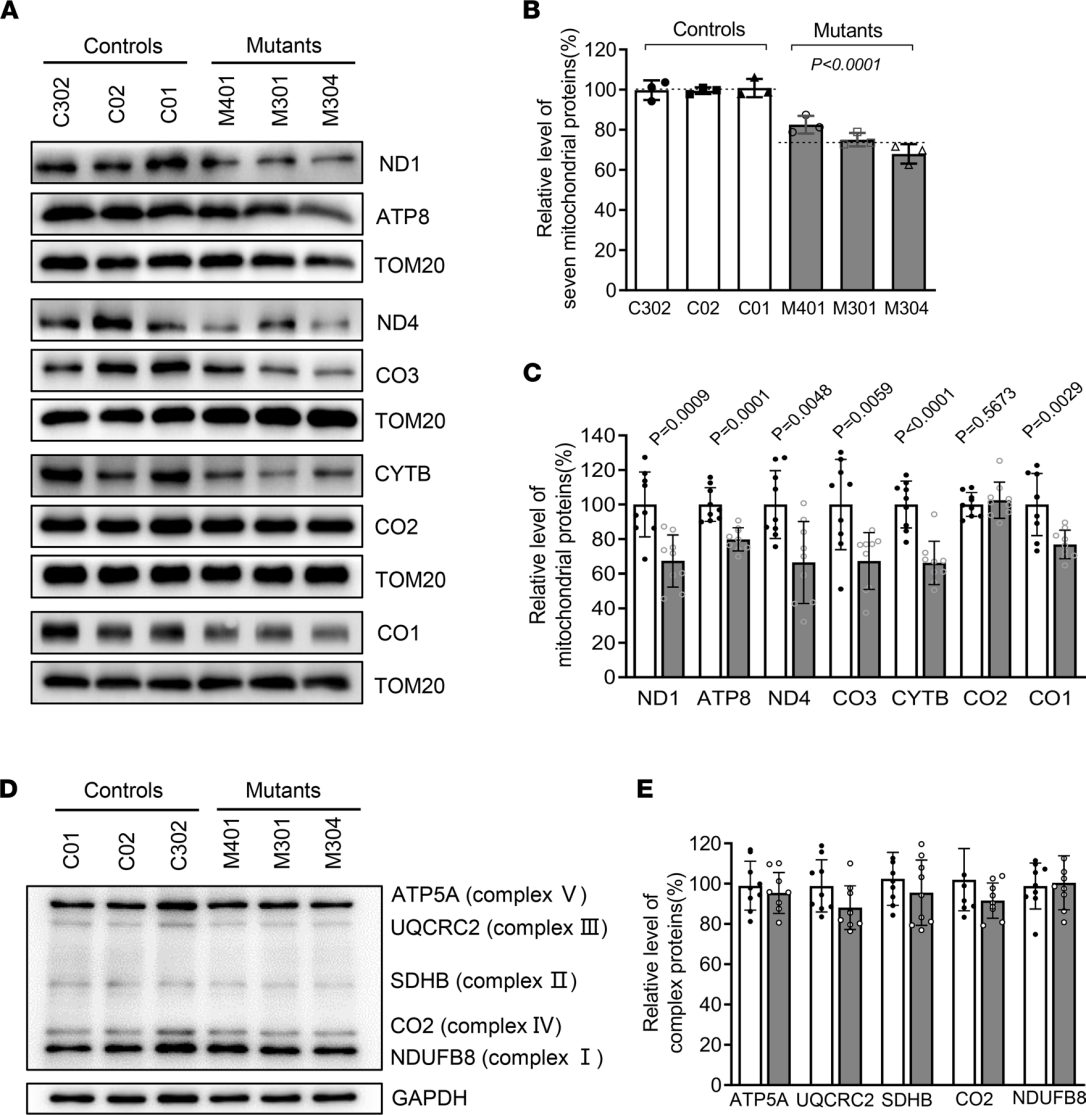
Western blot analysis of mitochondrial proteins and OXPHOS subunits. (**A**) Western blot analysis of mitochondrial proteins. In total, 20 μg of total proteins extracted from cells were denatured and electrophoresed on a 10% SDS–PAGE gel. The gel was electroblotted and probed with the primary Abs specific for ND1, ND4, CYTB, CO1, CO2, CO3, and ATP8, with TOM20 as the loading control. (**B** and **C**) Quantification of total mitochondrial protein levels and 7 polypeptide levels. The values for the mutant cell lines were expressed as percentages of the values for the control cell lines. (**D**) Western blot analysis of OXPHOS subunits. A total of 20 μg of total cellular protein were electrophoresed and hybridized with an Ab cocktail specific for subunits of each OXPHOS complex and with GAPDH as the loading control. (**E**) Quantification of the levels of ATP5A, UQCRC2, SDHB, CO2, and NDUFB8 in mutant and control cell lines. Solid column shows mutant cells. The data are expressed as the means ± SD based on 3 independent experiments. Student’s *t* test was performed to determine statistically significant differences.

As tRNA is a key molecule in decoding the genetic information in mRNA to generate amino acids (protein), the m.616T>C variant may impair mitochondrial translation. Western blot analysis was performed to measure the levels of 7 mtDNA encoding subunits involved in OXPHOS in various cell lines, with TOM20 used as the loading control (Figure 6A). The overall level of 7 mitochondrial translation products in mutant cell lines bearing the m.616T>C variant was 75.2% (*P* < 0.0001) that of the mean value measured in the control cell lines (Figure 6B). The levels of ND1 (67.3%), ND4 (66.4%), CO1 (76.8%), CO3 (67.3%), CYTB (66.2%), and ATP8 (79.8%) revealed various extents of reduced expressions in mutant cell lines, whereas the level of CO2 in the mutants was comparable with that of the controls (Figure 6C). Intriguingly, the decreased levels of polypeptides in the mutants did not correlate with either the number of codons or the proportion of phenylalanine residues in polypeptides (Supplemental Table 2). However, the m.616T>C did not diminish the levels of subunits encoded by nuclear genes (NDUFB8 in NADH: ubiquinone oxidoreductase; SDHB in succinate ubiquinone oxidoreductase; UQCRC2 in ubiquinol cytochrome c reductase; and ATP5A in H+ -ATPase) (Figure 6, D and E). Thus, the m.616T>C variant resulted in mitochondrial translation deficits.

## Discussion

Mitochondrial dysfunction plays an important role in the progression of kidney diseases (6). Recently, the term mitochondrial tubulointerstitial kidney disease (MITKD) was introduced to describe tubulointerstitial kidney disease caused by mtDNA variants, such as homoplasmic m.616T>C (MitoMap). In the present study, we identified cases in 3 families with isolated CKD and hyperuricemia associated with both homoplasmic and heteroplasmic m.616T>C in mt-tRNA^Phe.^ To date, several cases have been found with maternally inherited epilepsy and/or MITKD that were all caused by homoplasmic m.616T>C. It was first reported that 2 patients carrying the homoplasmic variant had severe epilepsy, which was the major symptom (13). In 2017, Connor et al. described 2 pedigrees harboring homoplasmic m.616T>C in which patients presented with encephalopathy and progressive renal failure (14). In 2020, Lorenz et al. published the case of a 5-year-old girl with chronic tubulointerstitial kidney disease and epilepsy, harboring homoplasmic m.616T>C in blood and urothelial cells, and her asymptomatic mother who carried the heteroplamic variant, presenting with 91% in blood, 82% in urothelial cells, and 86% on buccal swabs (19). Consistent with the high threshold of pathogenicity, the 35-year-old mother of family 2 (F2: II-4) harbored a mutant load of 68.21% in blood with normal indices in routine blood tests (Scr, 51 μmol/L; BUN, 4 mmol/L; and Uric acid, 255 μmol/L). Additionally, high loads of m.616T>C were discovered in different tissues from the asymptomatic 8-year-old sister (F3: III-2), 55.17% in blood, 77.25% in saliva, and 87.26% in urine. However, the mother of family 3 (F3: II-3), who was 38 years old, carried heteroplasmic m.616T>C, presenting with 76.27% in blood, 89.85% in saliva, and 94.5% in urine, showing that heteroplasmic m.616T>C can be pathogenic; furthermore, the high load of m.616T>C in the mother’s case led to CKD after more than 3 decades. However, neurological abnormality was not detected in any of these m.616 T>C carriers. Instead, 9 of the 14 patients presented with hyperuricemia, including the 3 probands. The age at onset of hyperuricemia in the fourth-generation of family 1 varied from 5–28 years, exhibiting an early-onset mitochondrial disorder as those reported elsewhere (20). Although hyperuricemia is an independent risk factor for CKD, the causal relationship between them in this study was unclear. As shown in Table 1, the variant caused CKD and led to ESRD in proband 1 when she was 6 years old, as reported previously (21), and in patient F1: II-2 at 51 years old, indicating the complexity of CKD progression.

mt-tRNA variants are major causes of human disease (18, 22, 23). Any variant that damages the mt-tRNA structure or disrupts aminoacylation, other modifications, or codon recognition can disrupt mitochondrial function. Swollen mitochondria were exhibited in mutant lymphoblast (Figure 2). Respirational deficits in mitochondria were evidenced by reduced OCRs, decreased mitochondrial ATP production, overproduction of ROS, and collapsed MMP, as reported elsewhere (17, 20); these, in turn, exacerbated mitochondrial dysfunction and caused cellular damage and progression of kidney disease (3, 24, 25). Alterations in both the structure and function of mt-tRNA^Phe^ were responsible for the mitochondria dysfunction due to m.616T>C. The variant m.616T>C abolished the highly conserved base pair (A31-U39) in the anticodon stem, which is critical for proper helical conformation during genetic code decoding (17, 26). The analysis of the MD simulation indicated that the disruption of the A31-U39 base pair led to reduced mt-tRNA^Phe^ stability, in agreement with the slower electrophoretic mobility and lower Tm (~2.4°C lower) of the mutant mt-tRNA^Phe^, C39. The altered structure contributed to the decrease in the steady-state level of mt-tRNA^Phe^. Here, a 46.4% reduction in the steady-state level of mt-tRNA^Phe^ was observed in mutant cells. Strikingly, the efficiency of aminoacylated mt-tRNA^Phe^ harboring C39 was enhanced, from a mean value of 50% in the controls up to 66% in the mutant cells, implying a possible compensatory effect on tRNA stabilization (27). Furthermore, aberrant tRNA metabolism, including altered aminoacylation and a shortage of tRNA^Phe^, led to impaired mitochondrial translation. A total of 13 structural subunits of the mitochondrial respiratory chain and ATPase (ND1–6; ND4L in complex I; CYTB in complex III; CO1, CO2, and CO3 in complex IV; and ATP6 and ATP8 in complex V) encoded by mtDNA are crucial for mitochondrial energetics (28). Markedly decreased levels of 6 mtDNA-encoded proteins were observed in mutant cells. In contrast to previous reports showing cells carrying mt-tRNA^Lys^ 8344A>G (29), the reduced levels of these polypeptides in mutant cells did not significantly correlate with the number or proportion of phenylalanine codons; this result was thought be a result of integration between lower amounts of mt-tRNA^Phe^ and enhanced mt-tRNA^Phe^ aminoacylation. The compensatory effects, such as enhanced aminoacylation and activated mitophagy to ameliorate mitochondrial function, may contribute to the high threshold of pathogenicity (30). Undoubtedly, these data support the idea that the pathogenic m.616T>C variant alters mt-tRNA^Phe^ stability and aminoacylation, leading to deficient mitochondrial translation and function.

In summary, this study shows that m.616T>C has a high pathogenic threshold, and both heteroplasmy and homoplasmy lead to isolated CKD and hyperuricemia. The pathogenic variant, m. 616T>C, alters both the structure and function of mitochondrial tRNA^Phe^, resulting in a shortage of mt-tRNA^Phe^ and enhanced aminoacylation, which lead to impaired mitochondrial translation and mitochondrial respiratory deficiency. These findings provide what we believe to be new insights into the pathogenesis underlying CKD due to mitochondrial variants.

## Methods

### Subjects.

Han Chinese family 1 was from accessed at the Children’s Hospital of Zhejiang University School of Medicine, Hangzhou, China. Families 2 and 3 were from the Children’s Hospital, Fudan University. Informed consent, blood, skin, saliva or urine samples, and photos were obtained from participating family members. Some family members were interviewed to understand both personal history and clinical manifestation. The concentration of Scr, BUN, and uric acid were tested in the laboratory of the Children’s Hospital. The probands underwent cranial MRI, electromyogram, renal ultrasound, and MRI scans during hospitalization.

### mtDNA analysis.

Genomic DNA was isolated from peripheral blood of participants using QIAamp DNA Blood Mini Kit (Qiagen, 51104). Entire mtDNAs of family members and 2 other control subjects (C01 and C02) were PCR-amplified in 24 overlapping fragments using sets of the L- and H-strand oligonucleotide primers as described previously (31). Each fragment was purified and then analyzed by direct sequencing. These sequencing results were compared with the updated consensus Cambridge sequence (NC_ 012920) (28). To analysis the presence and quantify the m.616T>C variant, the DNA fragments spanning the mt-tRNA^Phe^ gene were amplified by PCR using the following primers: 5′-CACCATCCTCCGTGAAATCA-3′ (forward) and 5′-AGGCTAAGCGTTTTGAGCTG-3′ (reverse), corresponding to the mtDNA template between positions 16,401 and 794. The 962 bp segment was subsequently digested with restriction enzyme Acl I since m.616T>C showed a site, “AACGTT”, for this restriction enzyme, and then separated in 3% agarose gel to distinguish the presence or absence of the homoplasmic m.616T>C variant. The quantification of mtDNA copy numbers from different subjects was carried out by real-time PCR, as detailed elsewhere (32). The relative mtDNA levels were calculated as the ratio of mtDNA/nDNA based on the obtained Ct values.

### Cell lines and culture conditions.

Immortalized lymphoblastoid cell lines were generated by transformation with the Epstein–Barr virus, as described elsewhere (33). A total of 6 cell lines were derived from 4 members of the Chinese family 1 (F1: III-1; F1: III-4; and F1: IV-1 harboring the m.616T>C variant and subject F1: III-2 lacking the variant) and 2 genetically unrelated control individuals (C01 and C02) belonging to the same mtDNA haplogroup (D4) of the proband (Supplemental Table 1). Cells were cultured in the RPMI 1640 medium (Invitrogen) with 10% FBS. Tubular epithelial cells were obtained from urine sediments of family 3 members (F3: II-3 and F3: III-1) harboring the m.616T>C variant and an unrelated control individual.

### Assessment of mitochondrial membrane potential.

Mitochondrial membrane potential was assessed with JC-10 Assay Kit-Flow Cytometry (Abcam) following the modified manufacturer’s recommendations.

### OCRs.

The OCRs were monitored by the XF-96 extracellular flux analyzer (Seahorse Bioscience), as detailed elsewhere (34). Each well had 10,000 cells seeded in XF96 plates. The OCR of each well was measured throughout programmed injections: Oligomycin (1 μM), Carbonyl cyanide 4-trifluoromethoxy phenylhydrazone (FCCP, 0.8 μM), Antimycin A (5 μM), and Rotenone (1 μM).

### ATP measurements.

The cellular and mitochondrial ATP levels were measured using the CellTiter-Glo Luminescent Cell Viability kit (Promega) following the manufacturer’s instructions.

### Mitochondrial superoxide measurements.

Quantification of mitochondrial superoxide was performed using MitoSOX-Red (Invitrogen) following the manufacturer’s instruction and the procedures detailed elsewhere (35). Briefly, approximately 1 × 10^6^ cells with or without H_2_O_2_ stimulation were harvested, resuspended in 5 μM MitoSOX reagent working solution, and then incubated at 37°C for 30 minutes in the dark. After washing, cells were analyzed by the BD-LSR II flow cytometer system (Beckton Dickson), with an excitation at 488 nm and emission at 529 nm. A total of 10,000 events were recorded in each sample.

### MD simulations.

The ASL of mt-tRNA^Phe^ (Figure 2A) was chosen for MD simulations. Crystal structure of human mt-tRNA^Phe^ within the tRNA^Phe^-PheRS complex (PDB ID: 3TUP) was chosen as a template to generate coordinates for the WT and mutant systems, with the coordinates of the backbone maintained and nt bases substituted to the mt-tRNA^Phe^ sequence using Chimera (36). To avoid the terminal fray, we changed the 2-terminal A-U base pairs to C-G base pairs. MD simulations were performed using the Amber 14 package (37) and the ff14SB force field (38). Each system was solvated with TIP3P water (39) in a cubic box. The Shake algorithm was applied for all hydrogen atoms (40). Energy minimizations were first performed to relieve unfavorable contacts, followed by equilibration steps to equilibrate the solvent fully. The system was first heated to 300 K in the NVT ensemble, followed by equilibration in the NPT ensemble (300 K, 1 bar). The production simulation was carried out with a time step of 2 fs, and each system was run up to 100 ns. MD trajectory was viewed with Visual Molecular Dynamics (VMD) software (41). Trajectory analyses were performed using the cpptraj program in Amber14 (42).

### UV melting assay.

The in vitro transcripts of WT and mutant mt-tRNA^Phe^ were produced by T7 RNA polymerase as previously described (43). The transcripts were diluted in the buffer containing 50 mM sodium phosphate buffer (pH 7.0), 50 mM NaCl, 5 mM MgCl_2,_ and 0.1 mM EDTA. Absorbance against temperature melting curves was measured at 260 nm with heating from 25°C–95°C at a rate of 1°C/min throughout an Agilent Cary 100 UV Spectrophotometer.

### mt-tRNA analysis.

Total mitochondrial RNAs were extracted from mitochondria which were isolated from lymphoblast cell line using a Ambion’s TOTALLY RNA kit (Thermo Fisher), as described elsewhere (44). For the tRNA mobility shift assay, 2 μg of RNAs were electrophoresed through a 10% PAGE, electroblotted, and hybridized with DIG-labeled oligonucleotide probes for mt-tRNAs.

For the Northern blot analysis, 2 μg of mitochondrial RNAs were separated by denaturing 10% PAGE with 8 M urea, electroblotted, and UV-crosslinked for the hybridization analysis of DIG-labeled oligodeoxynucleotide probes for mt-tRNAs and 5S rRNA as the loading control.

For the aminoacylation assays, total mitochondrial RNAs were purified under acid conditions, and 2 μg of mitochondrial RNAs were electrophoresed at 4°C by an acid (pH 5.0) 10% PAGE with 8 M urea in 100 mM sodium acetate buffer to separate the charged and uncharged tRNA. To further distinguish nonaminoacylated tRNA from aminoacylated tRNA, RNAs were deacylated by being heated at 60°C (pH 8.3) for 10 minutes and then analyzed with specific probes for mt-tRNA^Phe^, mt-tRNA^Tyr^, tournament, and mt-tRNA^Leu^ ^(CUN)^. Quantification of density in each band was performed as detailed elsewhere (18). For detailed oligonucleotide probes, see Supplemental Table 3.

### Western blot analysis.

A total of 20 micrograms of total proteins extracted from cells were denatured and electrophoresed on a 10% SDS-PAGE gel. The gel was electroblotted, and the PVDF membrane (MilliporeSigma) probed with the primary Abs interest. The image was visualized by Clinx ChemiCapture (Clinx Science Instruments), and the density of each band was quantified using ImageJ (NIH) software. A list of Abs used was provided in Supplemental Table 4.

### Immunofluorescence staining.

Tubular epithelial cells from urine samples were washed by PBS and fixed with 4% paraformaldehyde for 30 minutes, then permeabilized with 0.2% Triton X-100 for 15 minutes, and blocked in 5% BSA for 1 hour at room temperature. After blocking, cells were incubated with primary Abs overnight at 4°C. The next day, cells were coincubated with fluorescent secondary Abs for 1 hour and counterstained with DAPI (MilliporeSigma, D9542) for 8 minutes. Images were taken by Olympus Fluoview FV1000 microscopes. The primary Abs, anti-aquaporin 2 (anti-AQP2), anti-KIM1, and anti-NGAL, and the secondary Abs, Alexa Fluor 488 goat anti-mouse IgG and Alexa Fluor 594 goat anti-rabbit IgG, were listed in Supplemental Table 4.

### TEM.

Tubular epithelial cells were washed by PBS and fixed with 2.5% glutaraldehyde overnight at 4°C. After fixed with 1% OsO4 for 1–2 hours, cells were dehydrated by a graded series of ethanol (30%, 50%, 70%, 80%, 90%, and 95%) for 15 minutes and then dehydrated with absolute acetone twice for 20 minutes each. Cells were incubated in a 1:1 mixture of absolute acetone and resin for 1 hour, then transferred to a 1:3 mixture for 3 hours, and finally embedded in resin mixture overnight. The next day, samples were heated at 70°C for more than 9 hours, sliced by LEICA EM UC7 ultratome and stained by uranyl acetate and lead citrate for 15 minutes, respectively. Images were taken by the Hitachi Model H-7650 transmission electron microscope (Hitachi).

### Statistics.

Data were statistically analyzed using GraphPad Prism (Version 8.00) and presented by the mean ± SD. The unpaired, 2-tailed Student’s *t* test was used for comparing the outcomes of 2 groups. Neither randomization nor blinding was implemented for data analysis. A *P* value of less than 0.05 was considered statistically significant. The exact number of replicates and *P* values were described for each figure.

### Study approval.

All procedures followed were in accordance with approval from the Research Ethics Committees, the Children’s Hospital of Zhejiang University School of Medicine. All study participants or their guardians provided written informed consent before enrollment.

### Data and software availability.

The data set supporting the conclusions of this article is included within the article and its supplementary information files.

## Author contributions

PJ and JM conceived and supervised the project, designed the experiments, and interpreted the data; CX, JR, LT, QY, YC, HS, ZL, HF, and JM carried out the clinical evaluation and patients’ recruitment; CX, LT, YZ, JX, and FM performed biochemical and cellular experiments and data analysis; SW and WG carried out the genetic testing and data interpretation; LT, SW, and WG conducted the mutational sequencing and bioinformation analysis; XM and XC performed the MD analysis; and EYL, MG, PJ, and JM made the final version of the manuscript. All authors have read and approved the manuscript.

## Supplementary Material

Supplemental data

## Figures and Tables

**Table 1:**
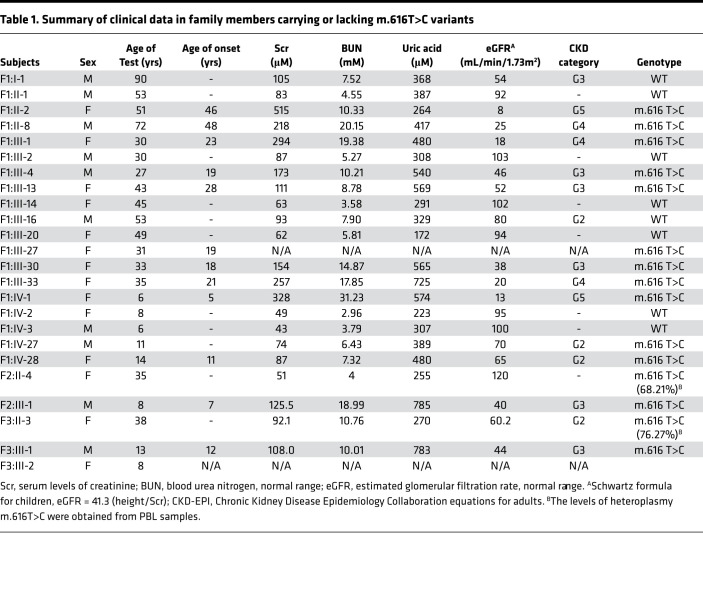
Summary of clinical data in family members carrying or lacking m.616T>C variants

## Version 1 04/26/2022

In-Press Preview

## Version 2 06/08/2022

Electronic publication
