# Supplementary material for: Heteroplasmic and homoplasmic m.616T>C in mitochondria tRNAPhe promote isolated chronic kidney disease and hyperuricemia
Source: JCI Insight. 2022 Jun 8;7(11):e157418. doi: 10.1172/jci.insight.157418 (PMC9220945; doi:10.1172/jci.insight.157418)
Supplement: Supplemental data [file jciinsight-7-157418-s080.pdf]

## Supplementary Materials

(3 figures and 4 tables)

1. Fig S1  Identification of the homoplasmic m.616 T>C in family 1
2. Fig S2  Heteroplasmy m.616 T>C and renal function by DTPA test
3. Fig S3  The expression levels of mitochondria shaping proteins

4.Table S1  Mitochondrial DNA variants and haplogroups in probands and three control subjects

5.Table S2  Usage of Phenylalanine codon in human mitochondrial genes.

6. Table S3  Oligonucleotides for mt- tRNA probes and primers for mtDNA copy number assay

7. Table S4  Key resources in experiments

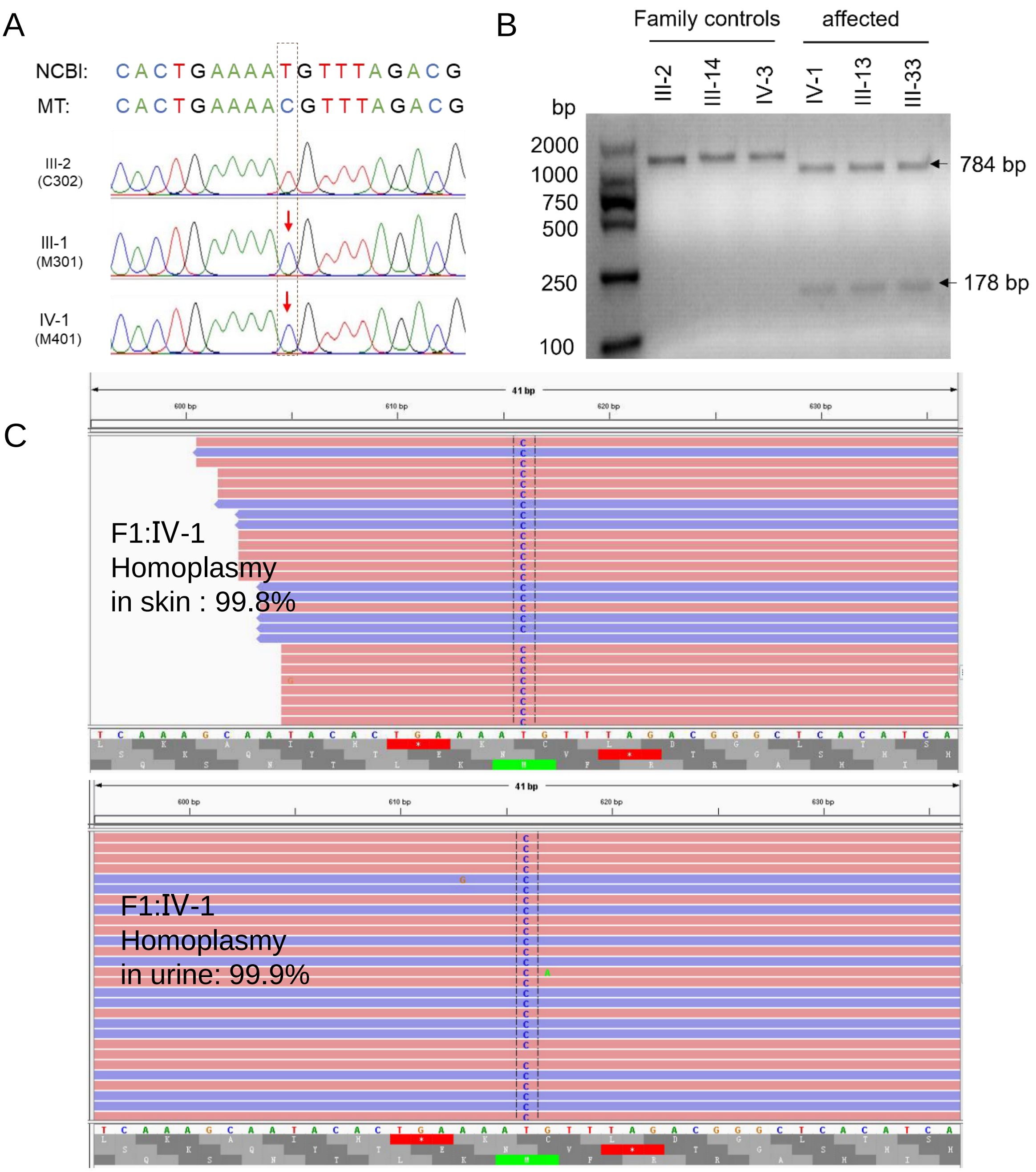


**Fig S1** ( related to Fig 1) Identification of the homoplasmic m.616 T>C in family 1.(A) Variant m.616T>C was confirmed by Sanger sequencing. Samples from unaffected control individual (father, III-2) and two affected individuals (mother, III-1 and proband, IV-1). (B) RFLP（restriction fragment length polymorphism） analysis of PCR fragments of samples from patients and control subjects, digested with enzyme AclⅠand then analyzed by electrophoresis through 3% agarose gel. The 962 bp segments with m.616 T>C was segmented into two fragments, 784bp and 178bp. (C) Homoplasmic m.616 T>C in skin and urine samples of proband by high-throughput sequencing.

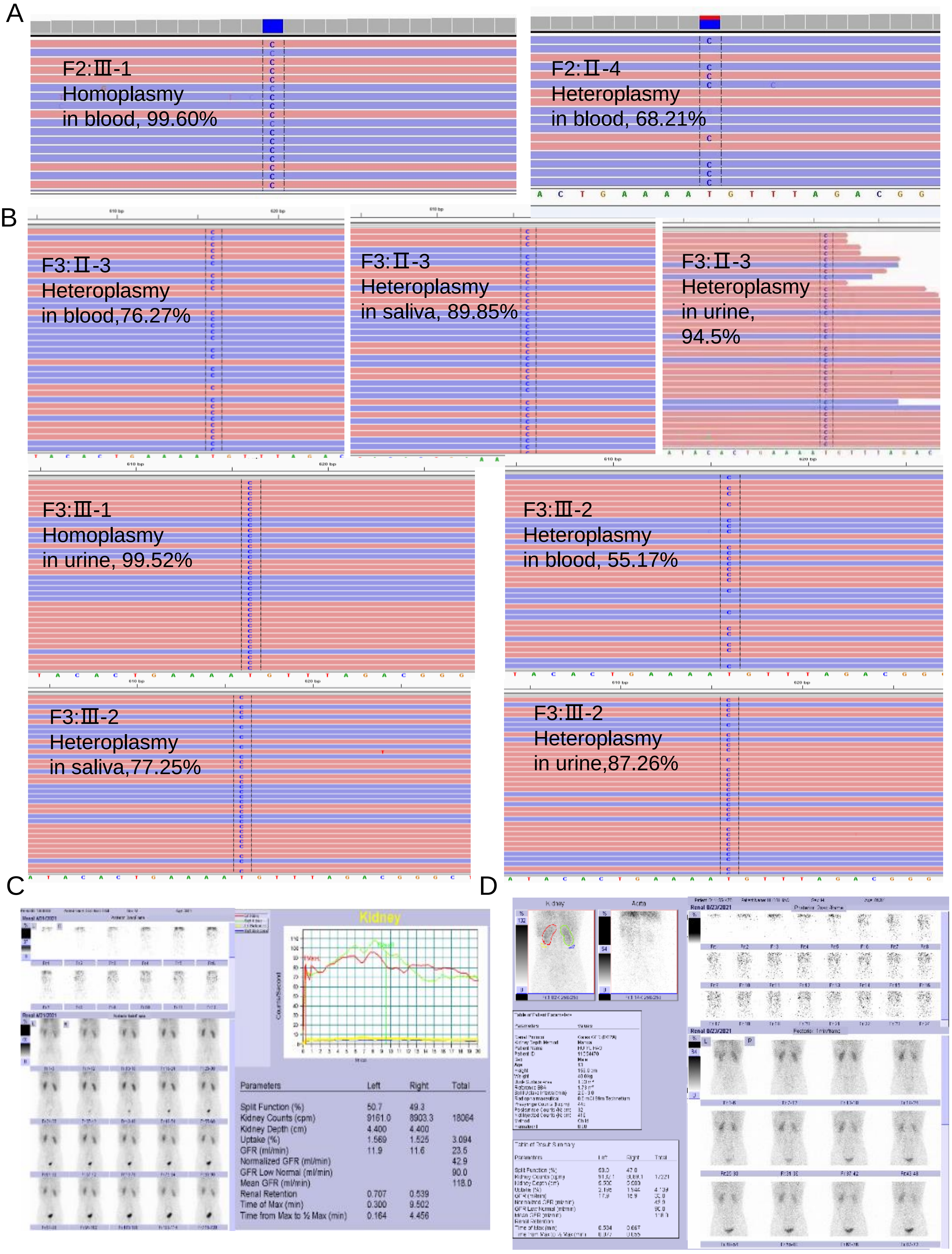


**Fig S2** ( related to Fig 1) Heteroplasmy m.616 T>C and renal function by DTPA test . Homoplasmic and heteroplasmic m.616T>C, comfirmed by high-throughput sequencing in different tissues. Heteroplasmic m. 616T>C was present in idividuals F2:II-4(A) ; F3:II-3 and patient F3:III-2 (B). DTPA test of proband 2 (C) and of proband 3 (D).

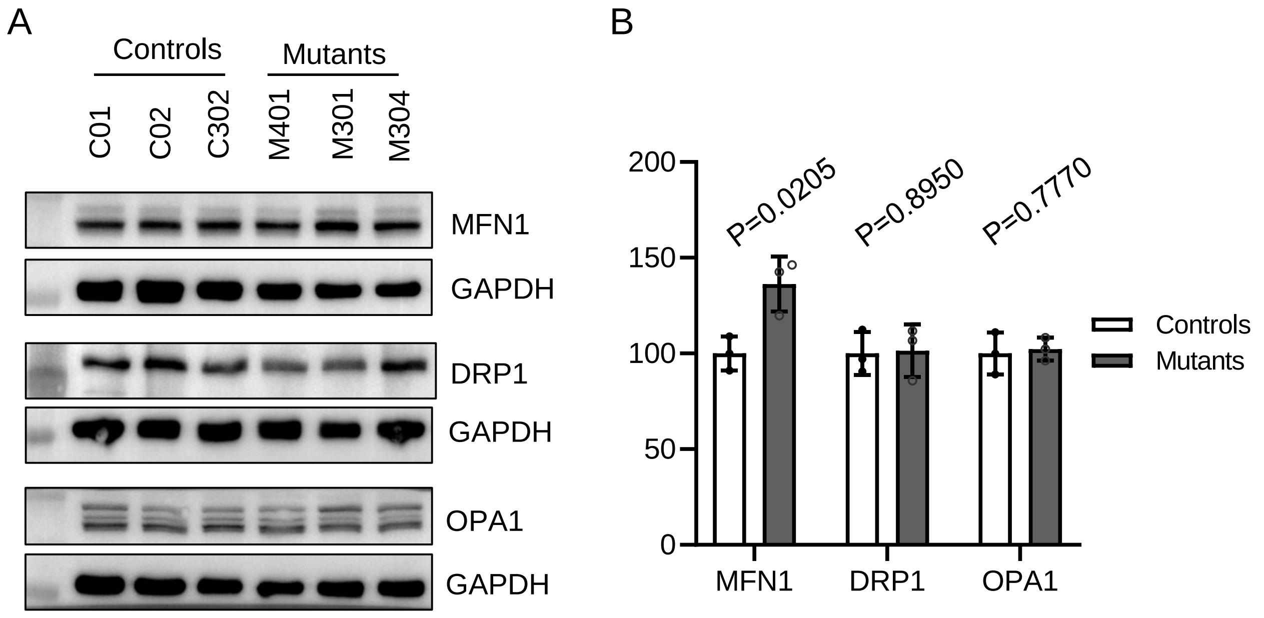


**Fig S3** ( related to Fig 2) The expression levels of mitochondria shaping proteins. (A) Western blotting assay for shaping proteins, MFN1, OPA1 and DRP1. (B) Quntification analysis.*, $P$<0.05 .

Table S1 Mitochondrial DNA variants and haplogroups in probands and three control subjects

| **Gene** | **Position** | **rCRS[a]** | **F1:Ⅳ-1 (D4)** | **F1:Ⅲ-2 (G4)** | **F2:Ⅲ-1 (F2)** | **F3:Ⅲ-1 (B5)** | **C01 (D4)** | **C02 (D4)** | **Previously reported[b]** |
|---|---|---|---|---|---|---|---|---|---|
| D-loop | 73 | A | G | G | G | G | G | G | Yes |
| | 103 | G | | | | A | | | |
| | 146 | T | | | | C | | | |
| | 152 | T | C | | | C | C | C | Yes |
| | 189 | A | | | | G | | | |
| | 204 | T | | | | C | | | |
| | 249 | A | | | - | | | | |
| | 260 | G | | A | | | | | Yes |
| | 263 | A | G | G | G | G | G | G | Yes |
| | 310 | T | CTC | CCTC | CTC | CTC | TC | TC | Yes |
| | 385 | A | | | | | G | | Yes |
| | 489 | T | C | C | | | C | C | Yes |
| | 16093 | T | | C | | C | | G | Yes |
| | 16129 | G | A | | | | A | A | Yes |
| | 16140 | T | | | | C | | | |
| | 16183 | A | | | | C | | | |
| | 16189 | T | | | | C | | | |
| | 16203 | A | | | G | | | | |
| | 16209 | T | | | C | | | | |
| | 16223 | C | T | T | | | T | T | Yes |
| | 16263 | T | | | | | C | | Yes |
| | 16278 | C | | T | | | | | Yes |
| | 16286 | C | T | | | | | | Yes |
| | 16291 | C | | | T | | | | |
| | 16304 | T | | | C | | | | |
| | 16311 | T | | | C | | | | |
| | 16325 | T | | C | | | | | Yes |
| | 16335 | A | | | G | | | | |
| | 16362 | T | C | C | | | C | C | Yes |
| | 16519 | T | | | C | C | C | C | Yes |
| tRNA[Phe] | **616** | **T** | **C** | | **C** | **C** | | | Yes |
| 12S rRNA | 709 | G | | A | | A | | | Yes |
| | 750 | A | G | G | G | G | G | G | Yes |
| | 1005 | T | | | C | | | | |
| | 1438 | A | G | G | G | G | G | G | Yes |
| | 1598 | G | | | | A | | | |
| 16S rRNA | 1824 | T | | | C | | | | |
| | 2526 | C | | | | | | A | |
| | 2706 | A | G | G | G | G | G | G | Yes |
| | 3010 | G | A | | | | A | A | Yes |

| | | | | | | | | | |
|---|---|---|---|---|---|---|---|---|---|
| | 3206 | C | T | | | | T | T | Yes |
| ND1 | 3951 | C | | | | T | | | |
| | 3970 | C | | | T | | | | |
| ND2 | 4483 | C | | | | G | | | |
| | 4703 | T | | | | C | | | |
| | 4769 | A | G | G | G | G | G | G | Yes |
| | 4883 | C | T | | | | T | T | Yes |
| | 5108 | T | | C | | | | | Yes |
| | 5178 | C | A | | | | A | A | Yes |
| $tRNA^{Ala}$ | 5601 | C | | T | | | | | Yes |
| CO1 | 6392 | T | | | C | | | | |
| | 6686 | C | | | | | T | | Yes |
| | 6737 | A | | G | | | | | Yes |
| | 7028 | C | T | T | T | T | T | T | Yes |
| | 7148 | T | C | | | | | | Yes |
| | 7170 | C | | | | T | | | |
| | 7185 | C | | | | | | A | |
| | 7186 | A | | | | | | C | |
| | 7389 | T | | | | | | C | |
| CO2 | 7600 | G | | A | | | | | Yes |
| | 7828 | A | | | G | | | | |
| | 8281 | C | | | | T | | | |
| | 8282 | C | | | | - | | | |
| | 8283 | C | | | | - | | | |
| | 8284 | C | | | | - | | | |
| | 8285 | C | | | | - | | | |
| | 8286 | T | | | | - | | | |
| | 8287 | C | | | | - | | | |
| | 8288 | T | | | | - | | | |
| | 8289 | A | | | | - | | | |
| ATP8 | 8414 | C | T | | | | T | T | Yes |
| | 8473 | T | C | | | | C | C | Yes |
| ATP6 | 8545 | G | | | A | | | | |
| | 8584 | G | | | | A | | | |
| | 8701 | A | G | G | | | G | G | Yes |
| | 8748 | C | | T | | | | | Yes |
| | 8829 | C | | | | T | | | |
| | 8860 | A | G | G | G | G | G | G | Yes |
| | 8930 | C | | | | T | | | |
| | 9128 | T | | | C | | | | |
| | 9144 | C | | T | | | | | Yes |
| CO3 | 9377 | A | | G | | | | | Yes |
| | 9540 | T | C | C | | | C | C | Yes |
| | 9575 | G | | A | | | | | Yes |

| | | | | | | | | | |
|---|---|---|---|---|---|---|---|---|---|
| | 9845 | T | | | | | C | | Yes |
| | 9950 | T | | | | C | | | |
| ND3 | 10310 | G | | | A | | | | |
| | 10398 | A | G | G | | G | G | G | Yes |
| | 10400 | C | T | T | | | T | T | Yes |
| ND4L | 10535 | T | | | C | | | | |
| | 10586 | G | | | A | | | | |
| ND4 | 10873 | T | C | C | | | C | C | Yes |
| | 11650 | A | G | | | | | | Yes |
| | 11719 | G | A | A | A | A | A | A | Yes |
| ND5 | 12338 | T | | | C | | | | |
| | 12361 | A | | | | G | | | |
| ND5 | 12705 | C | T | T | | | T | T | Yes |
| | 13563 | A | | G | | | | | Yes |
| | 13708 | G | | | A | | | | |
| | 13928 | G | | | C | | | | |
| ND6 | 14200 | T | | C | | | | | Yes |
| | 14494 | T | C | | | | | | Yes |
| | 14569 | G | | A | | | | | Yes |
| | 14668 | C | T | | | | T | T | Yes |
| CYTB | 14766 | C | T | T | T | T | T | T | Yes |
| | 14783 | T | C | C | | | C | C | Yes |
| | 14979 | T | C | | | | C | C | Yes |
| | 15001 | T | | | | C | | | |
| | 15043 | G | A | A | | | A | A | Yes |
| | 15223 | C | | | | T | | | |
| | 15301 | G | A | A | | | A | A | Yes |
| | 15326 | A | G | G | G | G | G | G | Yes |
| | 15508 | C | | | | T | | | |
| | 15662 | A | | | | G | | | |
| | 15813 | T | | | G | | | | |
| | 15824 | A | | | C | | | | |
| | 15851 | A | | | | G | | | |
| | 15852 | T | | | | G | | | |
| $tRNA^{Thr}$ | 15889 | T | | | | | C | | Yes |
| | 15927 | G | | | | A | | | |

[a] rCRS, revised Cambrige reference sequence (NC_ 012920).

[b] See the online mitochondrial genome database MITOMAP (https://www.mitomap.org/MITOMAP).

MtDNA haplogroups were analyzed by MitoTool (http://www.mitotool.org/), belonged to D4 (F1:IV-1, C01 and C02), G2 (F1:III-2, father), F2 (F2:III-1) and B5 (F3:Ⅲ-1) respectively. Probands: F1:IV-1, F2:III-1 and F3:III-1; Controls: F1:III-2, C01 and C02.

Table S2 Usage of Phenylalanine codon in human mitochondrial genes

| Complex | Protein | Number of amino acids (AA) | Number of Phenylalanine codons | Density of Phenylalanine codons (%) | Decrease level of mitochondrial proteins (%) |
|---|---|---|---|---|---|
| Complex I | ND1 | 318 | 16 | 5.03 | 32.7 |
| | ND2 | 347 | 15 | 4.32 | NA |
| | ND3 | 115 | 8 | 6.96 | NA |
| | ND4 | 459 | 20 | 4.36 | 33.6 |
| | ND4L | 98 | 3 | 3.06 | NA |
| | ND5 | 603 | 38 | 6.30 | NA |
| | ND6 | 174 | 8 | 4.60 | NA |
| Complex III | Cyt b | 380 | 24 | 6.32 | 33.8 |
| Complex IV | CO1 | 513 | 41 | 7.99 | 23.2 |
| | CO2 | 227 | 7 | 3.08 | 0 |
| | CO3 | 261 | 24 | 9.20 | 32.7 |
| Complex V | ATP6 | 226 | 13 | 5.75 | NA |
| | ATP8 | 68 | 2 | 2.94 | 20.2 |

Table S3 Oligonucleotides for mt- tRNA probes and primers for mtDNA copy number assay

| PRIMERS & PROBES | SEQUENCES (5'-3') |
|---|---|
| Primers | |
| 16S-F | CACCCAAGAACAGGGTTTGT |
| 16S-R | TGGCCATGGGTATGTTGTTAA |
| 18S-F | TAGAGGGACAAGTGGCGTTC |
| 18S-R | CGCTGAGCCAGTCAGTGT |
| Probes | |
| Phe | TGTTTATGGGGTGATGTGAGCCCGTCTAAA |
| Val | TCAGAGCGGTCAAGTTAAGTTGAAATCTCC |
| Leu-UUR | AGAAGAGGAATTGAACCTCTGACTGTAAAG |
| Met | TAGTACGGGAAGGGTATAACCAACATTTTC |
| Tyr | GGTAAAAAGAGGCCTAACCCCTGTCTTTAG |
| Ser-UCN | AAAGGAAGGAATCGAACCCCCCAAAGCTGG |
| His | AAATAAGGGGTCGTAAGCCTCTGTTGTCAG |
| Ser-AGY | GAGAAAGCCATGTTGTTAGACATGGGGGCA |
| Leu-CUN | ACTTTTATTTGGAGTTGCACCAAAATTTTT |
| 5S rRNA | GGGTGGTATGGCCGTAGAC |

Table S4. Key resources in experiments

| REAGENT or RESOURCE | SOURCE | IDENTIFIER |
|---|---|---|
| Antibodies | | |
| MTCO1 | Abcam | Cat# ab14705 |
| GAPDH | Abcam | Cat# ab8245 |
| total OXPHOS human WB cocktail | Abcam | Cat# ab110411 |
| MT-ND1 | Proteintech | Cat# 19703-1-AP |
| ATP8 | Proteintech | Cat# 26723-1-AP |
| LC3A | Cell Signaling Technology | Cat# 4599 |
| TOM20 | Abclonal | Cat# A19403 |
| MT-ND4 | Abclonal | Cat# A9941 |
| MT-CYB | Abclonal | Cat# A17966 |
| MTCO2 | Abclonal | Cat# A3843 |
| ATG12 | Abgent | Cat# AP1816a |
| MTCO3 | Novus Biologicals | Cat# NBP1-88063 |
| KIM1 | Abcam | Cat# ab228973 |
| NGAL | Abcam | Cat# ab125075 |
| AQP2 | Santa Cruz | Cat# Sc-515770 |
| Alexa Fluor 488 goat anti-mouse IgG | Abcam | Cat#ab150113 |
| Alexa Fluor 594 goat anti-rabbit IgG | Abcam | Cat#ab150080 |
| Goat anti mouse IgG(H+L) (HRP) | Beyotime | Cat# A0216 |
| Goat anti rabbit IgG(H+L) (HRP) | Beyotime | Cat# A0208 |
| Critical Commercial Assays | | |
| CellTiter-Glo® Luminescent Cell Viability Assay kit | Promega | Cat# G7571 |
| Lactate-Glo™ Assay | Promega | Cat# J5021 |
| MitoSOX™ Red Mitochondrial Superoxide Indicator, for live-cell imaging | Invitrogen | Cat# M36008 |
| JC-10 Mitochondrial Membrane Potential Assay Kit (Flow Cytometry) | Abcam | Cat# ab112133 |
| TRIzol reagent | Invitrogen | Cat# 15596018 |
| Genious 2X SYBR Green Fast qPCR Mix | Abclonal | Cat# RK21204 |
| T7 RiboMAX™ Express Large Scale RNA Production System | Promega | Cat# P1320 |
| Annexin V-FITC Apoptosis detection Kit with PI | BioLegend | Cat# 640914 |
| Chemicals and plasmids | | |
| Antimycin A | Sigma | Cat# A8674-50MG |
| Oligomycin | Sigma | Cat# 495455-10MG |
| FCCP | Sigma | Cat# C2920-10MG |
| Rotenone | Sigma | Cat# 45656-250MG |
| 2-DG | Sigma | Cat# D8375-1G |
| ATP | MCE | Cat# HY-B2176 |
